# A rare case of Epstein–Barr virus‐related plasmacytoma involving maxillary sinus mucosa

**DOI:** 10.1002/ccr3.959

**Published:** 2017-07-26

**Authors:** Julie D. Gibbs, Marino E. Leon, Kenia Liu, Johnny Nguyen, Ling Zhang

**Affiliations:** ^1^ Department of Pathology The University of South Florida Tampa Florida; ^2^ Department of Pathology The University of Florida Gainsville Florida; ^3^ Department of Hematopathology and Laboratory Medicine Moffitt Cancer Center Tampa Florida; ^4^ Department of Pathology John Hopkin All Children Hospital St Petersburg Florida

**Keywords:** Epstein–Bar virus, immunocompromised, plasmablastic lymphoma, plasmacytoma

## Abstract

Extramedullary plasmacytomas, Epstein–Bar virus (EBV) associated, are rarely encountered and usually have a fairly good clinical outcome. EBV+ plasmacytoma may cause a diagnostic dilemma as it phenotypically resembles an aggressive plasmablastic lymphoma (PBL). Herein, we report a unique case with maxillary EBV+ plasmacytoma from a 76‐year‐old immunocompetent individual.

## Introduction

Extramedullary plasmacytomas are mature monoclonal plasma cell neoplasms that usually involve the upper respiratory tract of immunocompetent individuals and are infrequently Epstein–Barr virus (EBV) related 1, 2. They typically follow a clinically indolent course and are usually treated with radiation therapy or limited chemotherapy regimens 1, 2. Plasmablastic lymphomas (PBL), on the other hand, are clinically aggressive B‐cell neoplasms, which usually lack B‐cell markers but express plasma cell markers 3, 4. PBL was first described in the oral cavity of human immunodeficiency virus (HIV)‐positive individuals and now is known to occur in other immunocompromised states and less often in immunocompetent individuals 3, 4. The Ki‐67 proliferative index of PBL is usually high and often >60% 3. EBV‐encoded RNA (EBER) is detectable in 75–100% of PBL but only in approximately 10% of plasmacytomas 4, 5. Unlike plasmacytomas, PBL has a higher 5‐year mortality rate despite aggressive treatment 3, 4. Clinically and histologically, plasmacytoma and PBL can be confidently distinguished, but a diagnostic dilemma may occur when a plasmacytoma is EBV positive and presents with unusual clinical or morphological features. An accurate diagnosis is critical for appropriate therapeutic decisions and prognosis.

## Case Report

Herein, we report a unique case of EBV‐related extramedullary plasmacytoma of maxillary sinus in an immunocompetent, HIV‐negative patient with characteristic clinical and histological features, excellent outcome, and long‐term follow‐up.

**Figure 1:**
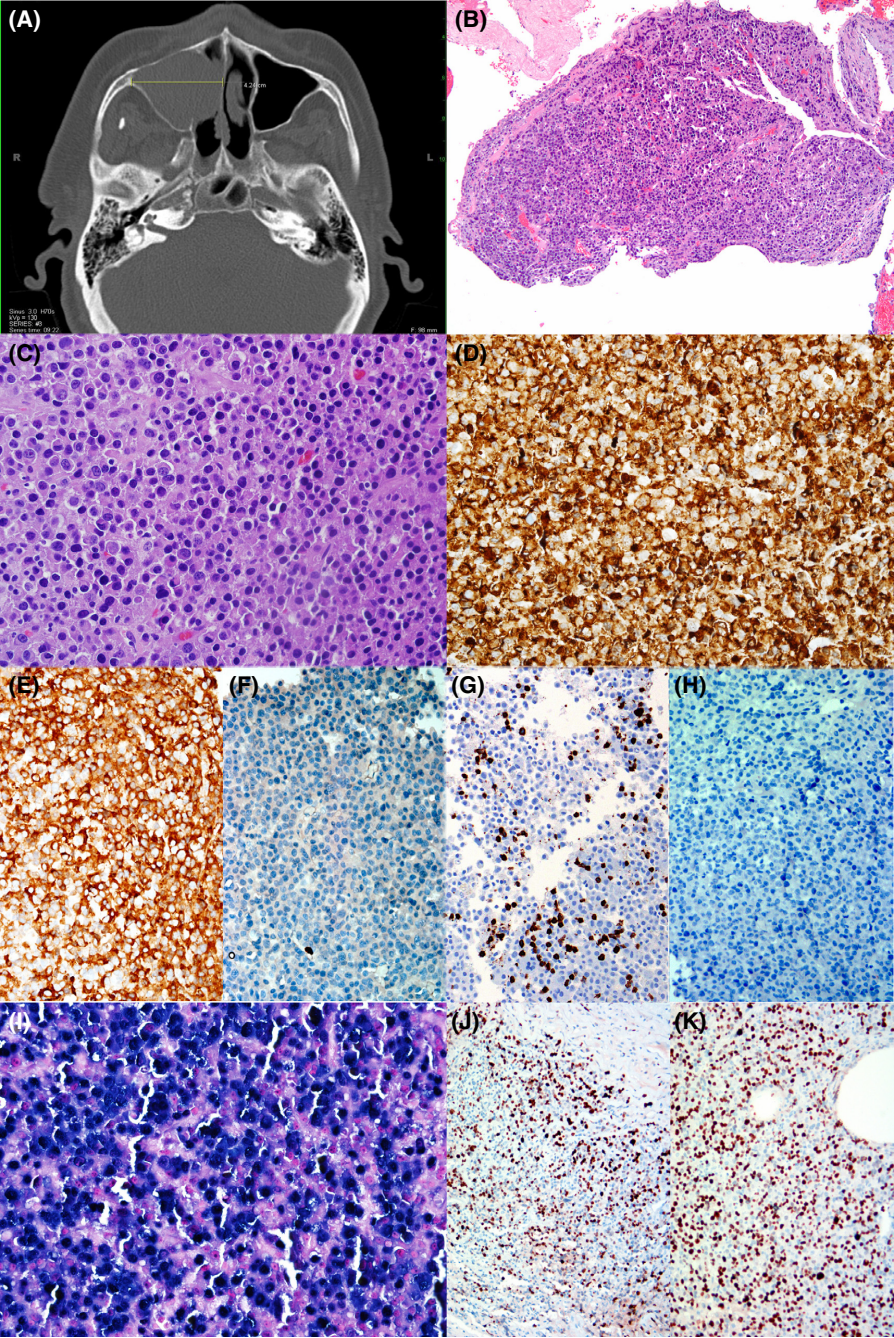
(A) CT scan of head showed 4.2‐mm mass located at the right nasal cavity. (B) Biopsy revealed mucosa‐covered polypoid mass (H&E, x40). (C) Medium power view of the H&E section showed numerous mature plasma cells with round to oval, eccentrically located nuclei with condense chromatin and abundant eosinophilic cytoplasm. Occasional enlarged plasma cells with hyperchromasia or binucleation are identified (H&E, x200). (C–H) Immunohistochemical stains highlight that plasma cells are strongly and diffusely positive for CD138 (D, x200) and kappa light chain (E, x100) and negative for lambda light chain (F, x100), CD8 (G, x100) and CD20 (H, x100). (I) In situ hybridization (ISH) with EBV‐encoded RNA probe revealed strong hybridization signals (x200). (J and K) Proliferation by Ki67 is slightly variable, ranging from 30% (J, x100) to focally up to 60% (K, x100).

A 76‐year‐old male patient was diagnosed with a T2N2bM0 squamous cell carcinoma of the left hypopharynx, which was treated with radiation and chemotherapy (completed December 2011). In March 2014, he presented again after two episodes of epistaxis. A positron emission tomography–computed tomography (PET/CT) scan at that time showed 18F‐fludeoxyglucose uptake in the right maxillary sinus, where a 4.2 × 4.4 × 3.9 cm expansile soft tissue mass had extended into the medial sinus wall (Fig. 1A). There was no general lymphadenopathy and hepatosplenomegaly by imaging studies. A sinus curettage was performed by an outside facility, consistent with “plasmacytoma.” The patient then presented to our center for further recommendations and treatment. A serum protein electrophoresis with immunofixation at that time showed an IgG‐kappa monoclonal gammopathy, whereas the urine protein electrophoretic pattern was within normal limits. A staging bone marrow biopsy in May 2014 was negative for malignancy, and a bone scan did not show any suspicious lytic lesions. Pathology slides, prepared outside our center, were reviewed to confirm the diagnosis.

The provided hematoxylin and eosin (H&E) sections showed a polypoid proliferation of predominantly mature plasma cells with a focal subset of atypical, less mature‐appearing plasma cells, which did not appear to infiltrate into the surrounding mucosa or submucosal soft tissue. Only a small subset of plasma cells showed slightly increased nuclear‐to‐cytoplasmic ratio and less condensed chromatin (Fig. 1B and C).

We analyzed the samples with a panel of immunohistochemical stains. The plasma cells were positive for CD138 (Fig. 1D), kappa light chain (Fig. 1E), CD56 (focal), CD117 (focal), and MYC (subset) and negative for lambda light chain (Fig. 1F), CD3, CD20 (Fig. 1H), and EBV‐LMP. Many CD8‐positive T cells were present in the background (Fig. 1G). The Ki‐67 proliferation index, by manual morphometric determination, was variable, ranging from 30% to 60%, with the higher index areas corresponding to areas of less mature‐appearing plasma cells (Fig. 1J and K). Chromogenic in situ hybridization studies showed the tumor cells to be positive for EBER (Fig. 1I). A MYC immunostain showed focal positivity (overall 5% of the total cellularity)(not shown). A FISH study using a *MYC* break‐apart probe, performed on a section of paraffin‐embedded tissue, revealed intact signals, indicating no *MYC* gene rearrangement. A bone marrow biopsy, together with flow cytometry and immunohistochemical stains, showed no evidence of clonal plasma cells. Cytogenetic and molecular studies performed on the bone marrow were normal (46,XY). The overall findings were consistent with an EBV‐related extramedullary plasmacytoma.

Treatment consisted of definitive radiation therapy (180–4500 cGy) to his right sinus over the months following, which he tolerated extremely well. A computed tomography scan performed in July 2016, 2 years after the initial diagnosis, showed no evidence of recurrent tumor. The patient is currently asymptomatic and doing well from a clinical standpoint, with no evidence of recurrent disease.

## Discussion

Only a handful of EBV‐positive plasmacytoma cases have been reported to date, with this disease occurring mostly in immunocompetent individuals 1, 2, 5. The entity has been associated with a background of CD8‐positive cytotoxic T cells 1. Loghavi et al. recently suggested the term EBV‐positive plasmacytoma in immunocompetent patients for these lesions 1. Unlike PBL, plasmacytomas typically have a low Ki‐67 proliferative index and lack MYC expression by immunohistochemistry 2, 3, 4. Although a few cases of plasmacytoma with a higher proliferative index and starry‐sky appearance have been reported, follow‐up suggests that these cases were either the initial presentation of myeloma, or could have been more appropriately classified as PBL 1, 4.

Although the exact role of EBV in pathogenesis remains unclear 2, 5, many believe that PBL may occur as a transformation from preexisting plasmacytoma after EBV infection 5. Ambrosio et al. recently reported a rare case of plasmablastic transformation of a preexisting plasmacytoma in an immunocompetent 74‐year‐old man initially diagnosed with an EBV‐positive, *MYC*‐negative plasmacytoma, who experienced multiple relapses with increasing proportions of EBV‐encoded RNA‐positive and MYC‐positive cells by immunohistochemistry (FISH negative) 5. A recent study from Motes‐Moreno's group showed genetic aberrations of *MYC* (approximately 46% translocation and 11% amplification) were detected in patients with PBL (36 cases) that lead to MYC protein overexpression 6. In contrast to most previously reported cases of EBV‐positive plasmacytomas, our case had a focally higher Ki‐67 proliferative index of up to 60% and had MYC expression (>5%) by immunohistochemistry, warranting a FISH study for *MYC* gene rearrangement. Negative *MYC* gene rearrangement favored a less aggressive clinical course and a lower likelihood of transformation to PBL. In addition, a subsequent EBV PCR study, performed on a peripheral blood sample, was negative. The patient responded well to local radiation and adjuvant chemotherapy and showed no recurrent disease to date, 2.5 years after initial diagnosis.

In conclusion, as shown in here, because extramedullary plasmacytomas are infrequently EBV related, clinically and histologically, a diagnostic dilemma regarding differentiation from PBL, which is typically EBV positive, can occur. A complete clinical and immunohistological investigation should help support an accurate diagnosis, which has substantial therapeutic and prognostic implications.

## Conflict of Interest

None declared.

## Authorship

JDG: collected the clinical and pathologic data and generated the manuscript. MEL and JN: reviewed clinical history and histology slides, provided representative images, and proofread the manuscript. KL: provided additional FISH study to prove no MYC gene rearrangement in the case. LZ: supervised and instructed JDG how to write the case report according to the journal's instruction, obtained the patient's consent, provided additional information and responses per reviewers' request, and revised the manuscript.
